# Submuscular gluteal abcess: An unusual presentation of rare sacral tuberculosis

**DOI:** 10.1016/j.ijscr.2018.11.046

**Published:** 2018-11-24

**Authors:** Yoshi Pratama Djaja, Phedy Phedy, Jamot Silitonga, Didik Librianto, Ifran Saleh

**Affiliations:** a Department of Orthopaedic and Traumatology, Fatmawati General Hospital, Jl. RS Fatmawati No. 1, Cilandak, South Jakarta, 12430, Indonesia; b Department of Orthopaedic and Traumatology, Cipto Mangunkusumo General Hospital - Faculty of Medicine Universitas, Jl. Pangeran Diponegoro No. 71, Salemba, Central Jakarta, 10430, Indonesia

**Keywords:** Sacral tuberculosis, Gluteal abscess, Spinal tuberculosis

## Abstract

**Introduction:**

Both gluteal abscess and sacral tuberculosis are rare entities in spinal tuberculosis cases. Even in endemic country, this atypical presentation may be the cause of delayed diagnosis and treatment.

**Presentation of case:**

A 51-year-old woman was admitted with painless massive lump on both of her thighs that have been enlarging for the past 6 months. She had a history of previous tuberculosis treatment. From the MRI examination submuscular gluteal abscess, which was an extension of the sacral tuberculosis, were found. Open debridement and biopsy were performed, which confirmed the suspicion of tuberculosis. Oral anti tuberculosis drugs were administered after. There was no recurrence and complication at the final follow up.

**Discussion:**

Cold abscess formation is common in spine tuberculosis however the formation of gluteal abscess as the extension of sacral tuberculosis is rare. Although MRI's specificity in determining the underlying cause is poor, it has a great role not only determining the location and size of the lesion, but also to describe anatomical pathophysiology of the abscess dissemination from sacral tuberculosis.

**Conclusion:**

Despite the limitation of the study and the rarity of this case, tuberculosis should be made as the main differential diagnosis for atypical sacral lesion that occurs with submuscular gluteal abscess.

## Highlights

• Gluteal abscess and sacral tuberculosis are rare entities in spine tuberculosis, as to our knowledge; this is the second case report about it.
• MRI has a great role to describe anatomical pathophysiology of the abscess dissemination from sacral tuberculosis.
• Sacral tuberculosis should be made as the main differential diagnosis for atypical sacral lesion that occurs with submuscular gluteal abscess.

## 1 Introduction

Tuberculosis is a continuing problem especially in developing countries despite the availability of effective chemotherapy. Around 1–2% of tuberculosis patients have involvement of the skeletal system and 50% of them involve the spinal column [1]. Most spinal involvement of tuberculosis occurs in lumbar spine region and some of those are also presented with psoas abcess [1,2].

On the other hand, the incidence of both gluteal abscess and sacral tuberculosis are extremely rare. The previous studies on sacral tuberculosis were limited in case reports [[3], [4], [5]] and few case series [6,7]. We describe a case of 51-year-old woman with sacral tuberculosis and bilateral massive submuscular gluteal abscesses. To our knowledge, this is the second case of sacral tuberculosis that presented with submuscular gluteal abscess, which gave us a diagnostic challenge and treatment dilemma. The present case has an interesting magnetic resonance imaging (MRI) pattern that described the anatomical pathophysiology of abscess dissemination in the sacral and gluteal region. Our case report has also been reported in line with the SCARE criteria [8].

## 2 Case report

A 51-year-old woman presented with a massive painless lump on both of her thighs that had been enlarging for the past 6 months. The patient denied any history of trauma, manipulation, or injection around the lump before. She was otherwise healthy despite her lumps. However, she had a history of lymph node tuberculosis on her neck about 25 years before and underwent tuberculosis chemotherapy regiment for about six months.

**Fig. 1:**
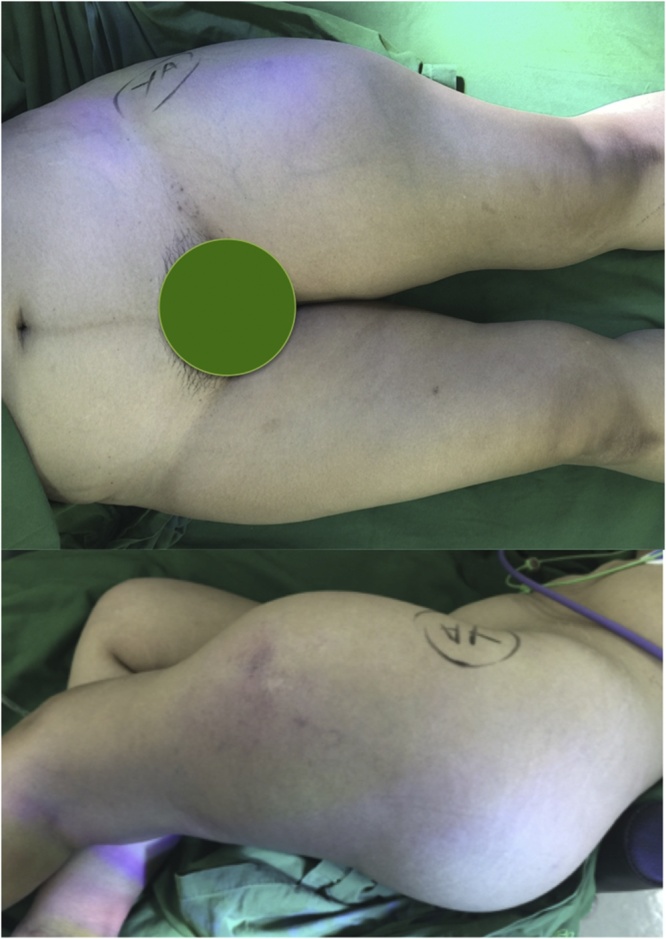
Clinical presentation of massive gluteal abscess that extended distally to anterolateral femur.

On the local physical examination, we found a painless non-mobile distention on her gluteal and upper femoral region bilaterally with some fluctuation and cystic consistency on palpation of the mass. The initial largest diameter of her thigh was 60 cm on the left and 45 cm on the right. There was no signs of inflammation, sinus or fistula around her thighs and buttock, or any remarkable signs on physical examinations (Fig. 1). Laboratory examinations however, showed elevated level of ESR and CRP. Mantoux test were inconclusive due to previous infection of tuberculosis. Radiological examination showed no signs of abnormality besides the expanding soft tissue shadow especially on her left femur region.

**Fig. 2:**
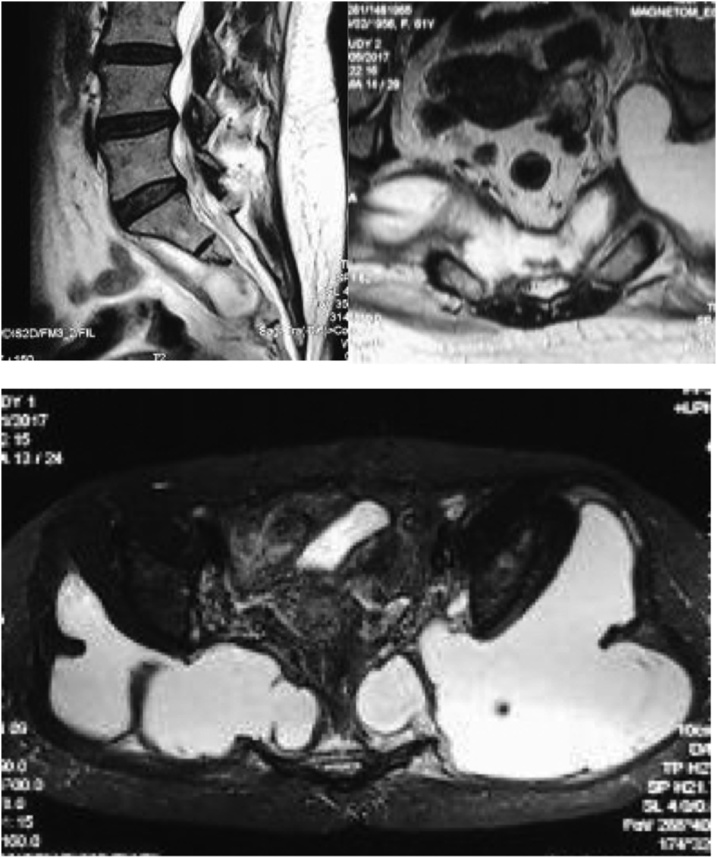
(a) T2-weighted sagittal MR images of sacral spine showing presacral abscess on distal sacral segment; (b) T2-weighted axial MR images on sacral spine (cut on the level of red line in image on the left) showing extension of the abscess to insertion of piriformis; (c) T2-fat suppressed axial images on pelvic showing further lateral extension on the abscess.

MRI examinations were then performed over the lumbosacral and pelvis region. Sagittal T2 weighted MR images of the sacrum showed destruction on anterior lower sacral segments, with hyperintense anterior lesion and presacral abscess. Axial T2 weighted images confirmed sacral body destruction and extension of the hyperintense lesion that involved the insertion of piriformis muscle (Fig. 2a and b).

Pelvic axial fat-suppressed (FS) T2 weighted images gave another extended view of the lesion, showing lateral extension of the lesion over the posterior ilium that also extended to superior and inferior filling the gluteal compartment beneath the gluteus maximus and tensor fascia lata (Fig. 2c). Involvement of the piriformis muscle and gluteus medius were confirmed at the coronal FS-T2 images of proximal femur, in which there was a hyperintese bony lesion at the tip of greater trochanter.

**Fig. 3:**
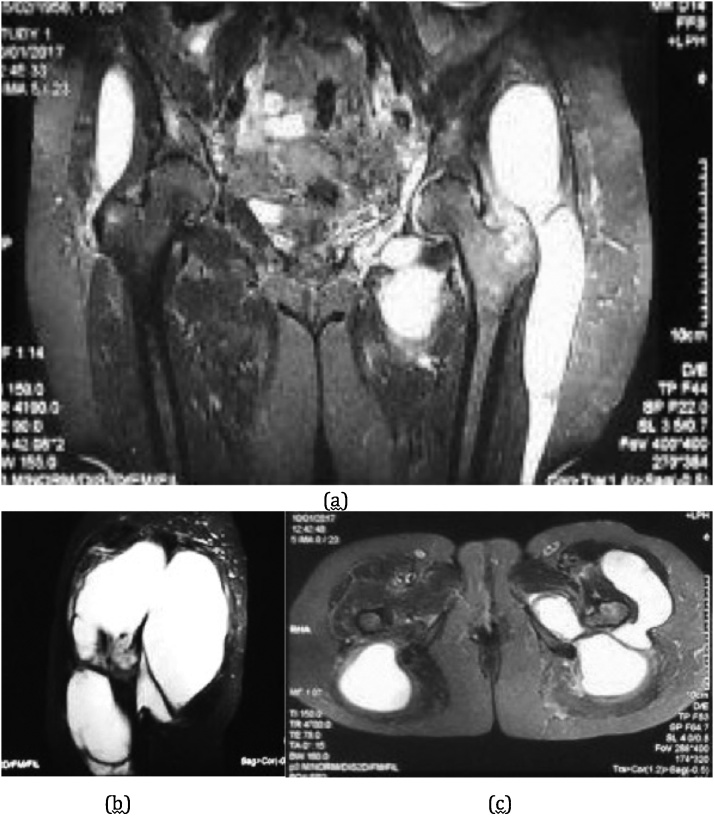
(a) Coronal T2-FS MRI images of pelvic and bilateral femur showing extension of the abscess distally around the greater trochanter beneath the gluteus maximus muscle and tensor fascia lata; (b) Sagittal T2-FS MRI images centered around the greater trochanter; (c) Axial T2-FS MRI cut on the level of proximal femur just distal to the lesser trochanter showing pattern of abscess extension distally.

The abscess also extended distally through the space around the greater trochanter between the vastus lateralis and tensor fascia lata without any intracapsular involvement of the hip joint (Fig. 3a). Distal extension of the abscess reached the level of midshaft femur beneath the hamstring and tensor fascia lata muscle (Fig. 3c).

**Fig. 4:**
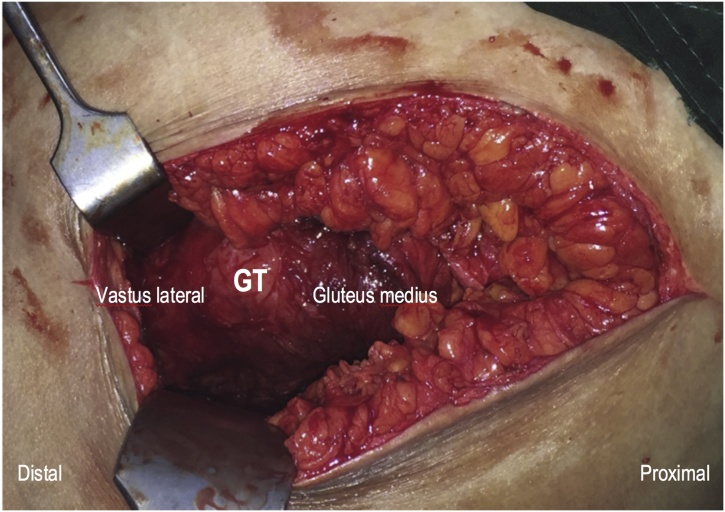
Intact muscular structure of vastus lateralis and gluteusmedius, which are usually preserved in tuberculosis abscess.

Due to the previous history of tuberculosis and the endemic nature of the disease, we started anti-tuberculosis treatment (ATT) initially for about 2 weeks. Serial laboratory examination of ESR and CRP showed remarkable decrease, which substantiates our working diagnosis. Surgical debridement and biopsy was performed. A curved incision centered on the left greater trochanter was used since it was the convergence point of the abscess on MRI images (Fig. 3b). Superficial dissection of posterior approach of the hip were used and after dissecting the gluteus maximus muscle, sero-purulent liquid discharged from the plane. Further debridement was performed and about 2.7 liters of pus were evacuated. There was intact muscular structure around the greater trochanter as well as the short external rotator thus the debridement were not extended to the hip joint (Fig. 4). An additional debridement through posterior midline incision through the multifidus muscle around the sacral region were also performed but turned out negative. During the wound closure, a submuscular drain was put and minimal production was found during the first two days. The patient was discharged and started her antituberculosis drug regime due to high suspicion of tuberculosis.

On the two weeks follow up, the surgical wound healed without any complication and there were no signs of recurrent fluid collection. Despite negative result on the culture result, the histopathological examination showed a necrotic tissue with appearance of epitheloid cells, lymphocytes, histiocytes, and Langhans multinucleated giant cells which was in accordance with chronic inflammation due to tuberculosis. Neither recurrence nor complication was found after 6 months follow up.

## 3 Discussion

Tuberculosis of the spine is major health problem especially in endemic regions. The current incidence of spine tuberculosis is around 50% of all skeletal tuberculosis cases. From previous epidemiological studies, sacral spine involvement only accounts for 0–6.3% of spine tuberculosis cases [9]. The exact explanation for the low incidence of sacral tuberculosis are not clearly described in previous literature. However, the anatomical vascularity of the sacrum, local oxygen level and dissemination pattern of the mycobacteria from the primary foci, hypothetically, have some contributions for its rarity [10]. The rarity of this subgroup of disease produced some difficulty especially in diagnosing the exact etiology of the symptoms, as it may mimic other diseases such as pyogenic infection or malignancy. Gluteal abscess, for example, which is one of the rarest presentations of spinal tuberculosis, is more often associated with pyogenic infection in perianal region [11].

Cold abscess formation is common in spine tuberculosis, with a prevalence of 55–86%. In sacral spine, presacral abscess is the most common presentation, which accounts around 40% of cases [7]. Sacral tuberculosis presenting as gluteal abscess, however, is very rare. Kumar presented the first case of submuscular gluteal abscess in a 3-year-old-female-child as the extension of her sacral tuberculosis [12]. Puthezhath reported another tuberculosis case of lumbar spine with the gluteal abscess as the only symptoms [10]. Based on CT evaluation on his case, the location of the abscess were very superficial, which was not suitable with the anatomical extension nor proposed pathophysiology of the disease.

Magnetic resonance imaging is the most sensitive diagnostic procedure, however it has low specificity in diagnosing sacral tuberculosis as it may be misinterpreted as other infections or malignancy. Despite providing an excellent evaluation of the disease extension, MRI alone cannot determine the exact cause of the abscess. Histopathologic examination is the current gold standard in determining the exact diagnosis, provided that an accurate tissue biopsy was performed.

Evaluation the extent of the abscess and the involved structure on MRI will provide not only the information about the site of biopsy but also about the probable pathophysiology of the disease. Based on the anatomic hypothesis on MRI images, the cold abscess over anterior vertebral bodies on S2–S4 may spread along the prevertebral space through the origins of piriformis muscles bilaterally to their insertions on the greater trochanter, which would allow the pus to spread beneath the gluteus maximus proximally and tensor fascia lata distally following the course of least resistance through intermuscular planes [13]. Inexistence of separating fascia around the gluteal compartment allowed the fluid to spread massively and form a massive gluteal abscess.

Another proposed theory of the gluteal abscess formation is by hematogenous spread along the branch of the aorta from the lumbar or sacral spine [14,15]. Based on this theory, the final presentation of the cold abscess may extent to the (a) ischiorectal fossa, (b) submuscular gluteal abscess, (c) psoas sheath, (d) lumbo-dorsal region (Petit’s triangle) or even more distally to the medial side of the thigh, popliteal fossa or medial side of Achilles tendon [14]. However, this scenario is unlikely because the absence of systemic infection and the patient’s immunocompetent status. The MRI images also shown that the extension of the abscess was mostly occurred on the posterior and lateral side of the thigh, which was not in accordance with the previous hematogenous theory.

It is the current general consensus that tuberculosis is actually a medical disease [20]. Anti-tuberculosis drugs especially isoniazid and ofloxacin are more than capable to permeate into the cold abscess. Despite having their maximum concentration significantly lower than the serum concentration, it still reached the level beyond their respective MIC and disappeared more slowly compared with that in the serum. However, in massive abscesses like in our case, the fluid’s volume within may dilute the anti-tuberculosis drug and reduce their concentration below the MIC. Thus, surgical debridement and abscess evacuation are indicated in the presence of massive abscess, despite lack of general agreement about the actual cut-off on the abscess volume.

Percutaneous drainage of the abscess is usually recommended in most spine tubercular abscess without any indication for spinal stabilization [2,16]. Open debridement and biopsy are warranted in several cases, where the diagnosis is still unclear thus adequate tissue sample has to be obtained. Estimated volume of the abscess is also a consideration in determining the method of evacuation. Although there isn’t any general consensus about the cut off point, the abscess in our case is massive and too extensive to perform a percutaneous drainage. Dinc et al. [17], performed an analysis of image guided percutaneous drainage of iliopsoas tuberculous abscess in 26 abscesses. In their series, the drainage volume was ranged from 85 to 1450 mL (mean volume 321 mL), which are significantly much lower than our case. Other factors such as delayed presentation of the disease may exhibit formation of granulation tissue, which will require more aggressive approach [18]. Optimal debridement will improves the pharmacokinetic effectiveness of anti-tuberculosis drugs [19,20].

For patients presenting with submuscular gluteal abscess, consideration of spine tuberculosis as the potential source, is mandatory especially in developing or endemic countries. Early diagnostic and management may prevent further morbidity and improves the patient’s outcome.

## 4 Conclusion

Tuberculosis should be the first differential diagnostic in atypical presentation of sacral lesions and submuscular gluteal abscess. MRI examination combined with a structural anatomical analysis is a very useful in evaluating the abscess dissemination process and should be performed in regular basis.

## Conflicts of interest

None.

## Funding source

None.

## Ethical approval

Ethical approval was exempted by our institution (Fatmawati General Hospital Ethical Committee).

## Consent

We confirmed that written informed consent were obtained from the patient.

## Author contribution

All author (YPD, JS, Ph, DL, IS) participate in study concept, data interpretation, writing and editing the paper.

First author (YPD) were also responsible in data collection, interpretation, writing and editing the paper.

The second and third authors (JS and Ph) were the main attending for the patient, and performing the diagnosis, surgery and follow up.

Fourth and Fifth authors (DL and IS) were responsible in study concept, data interpretation and editing the paper.

All authors contributed in proposal preparation, data collection and follow up, and final manuscript preparation.

## Registration of research studies

N/A.

## Guarantor

First/ Corresponding author: Yoshi Pratama Djaja.

## Provenance and peer review

Not commissioned, externally peer reviewed.
